# “Mushroom in the heart”: a *Volvariella volvacea* infective endocarditis case report

**DOI:** 10.3389/fcvm.2025.1643975

**Published:** 2025-09-15

**Authors:** Yunhan Mao, XinPei Liu, ChaoJi Zhang, Jun Zheng

**Affiliations:** Department of Cardiac Surgery, Peking Union Medical College Hospital, Beijing, China

**Keywords:** *Volvariella volvacea*, infective endocarditis, invasive fungal infections, allogeneic HSCT, fungal endocarditis surgery

## Abstract

*Volvariella volvacea (V. volvacea)*, an edible mushroom, may act as a pathogenic agent causing invasive fungal infections (IFIs) in immunocompromised patients. We present a 38-year-old male with persistent high fever post-allo-HSCT. Plasma mNGS revealed rising *V. volvacea* DNA loads (1,137 copies/μl). Intravenous antifungal therapy was initiated upon the diagnosis of IFI. Transthoracic echocardiography showed a 4 × 1 cm left atrial vegetation, with enhanced CT confirming multiorgan septic emboli (brain and kidney). PET/CT revealed a left atrial vegetation originating from a right lung infectious lesion, spreading contiguously into the left atrium via the pulmonary vein. Urgent vegetation resection was performed, followed by continued intravenous antifungal treatment. At the 5-month follow-up, the patient was afebrile with negative mNGS, completely resolved pulmonary lesion, and an improved quality of life. This case highlights the potential value of surgical-targeted antifungal therapy for fungal endocarditis and suggests practical principles: including mNGS-guided diagnosis, urgent surgical excision, long-term optimized antifungal therapy, and regular follow-up surveillance of the residual infected lesion.

## Introduction

Invasive fungal infections (IFIs) pose a significant threat to immunocompromised patients, contributing to elevated mortality (1). Among allogeneic hematopoietic stem cell transplantation (allo-HSCT) recipients, the 1-year mortality rate for IFIs ranges from 36.0% to 72.0% (2). Cases of aspergillus endocarditis after HSCT were reported (3, 4). *V. volvacea* is an edible mushroom and traditionally regarded as non-pathogenic. The first case of IFIs caused by *V. volvacea* was reported in 2010 (1). We present a case of *V. volvacea* endocarditis in a patient following allo-HSCT, which was successfully managed through a combination of surgical intervention and antifungal therapy.

## Case report

**Figure 1:**
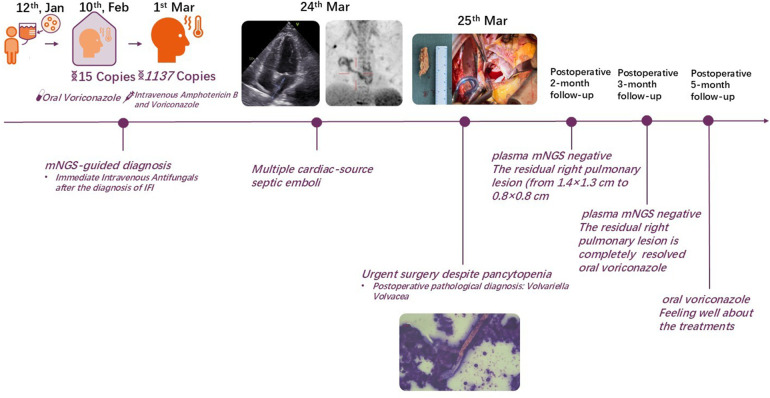
Treatment timeline for the present case.

**Figure 2:**
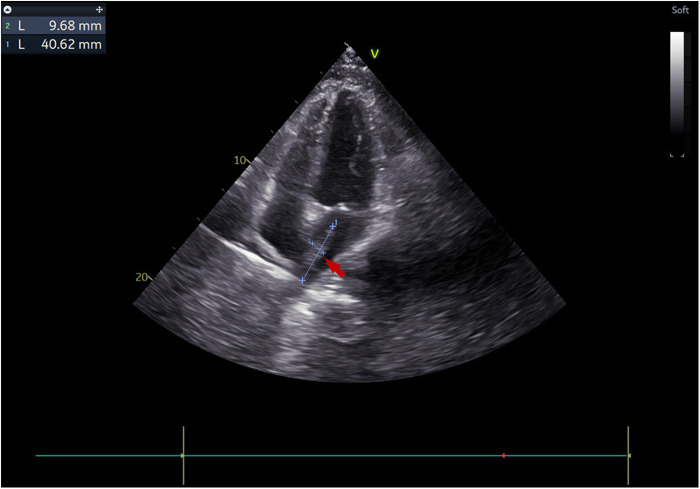
Transthoracic echocardiography visualized a 4 × 1 cm pedunculated vegetation (arrow) in the left atrium in the apical four-chamber view.

The treatment timeline for this patient is shown in (Figure 1). A 38-year-old male with a history of autoimmune lymphoproliferative syndrome (ALPS) and secondary hemophagocytic lymphohistiocytosis (HLH) underwent allo-HSCT on January 12, 2025. Post-HSCT course in the protective isolation unit was uneventful. Following isolation unit discharge on February 10, he developed persistent fever. Plasma metagenomic next-generation sequencing (mNGS) detected *V. volvacea* DNA (15 copies/μl). Oral voriconazole was initiated on the same day for suspected fungemia. On March 1, his fever escalated to 39°C. Repeat mNGS testing reveals a marked increase in fungal load (1,137 copies/μl). He was then diagnosed with IFIs and treated with intravenous amphotericin B and voriconazole starting March 3. Fever control was achieved in 2 days. On March 11, the peripheral blood fungal load reduced to 2 copies/μl. On March 15, the patient experienced a new-onset mid and lower back pain. A contrast-enhanced CT was performed to revel multiple emboli in the right parietal lobe of the brain and both kidneys. Transthoracic echocardiography (TTE) demonstrated a 4 × 1 cm left atrial vegetation arising from the right inferior pulmonary vein (RIPV) (Figure 2).

**Figure 3:**
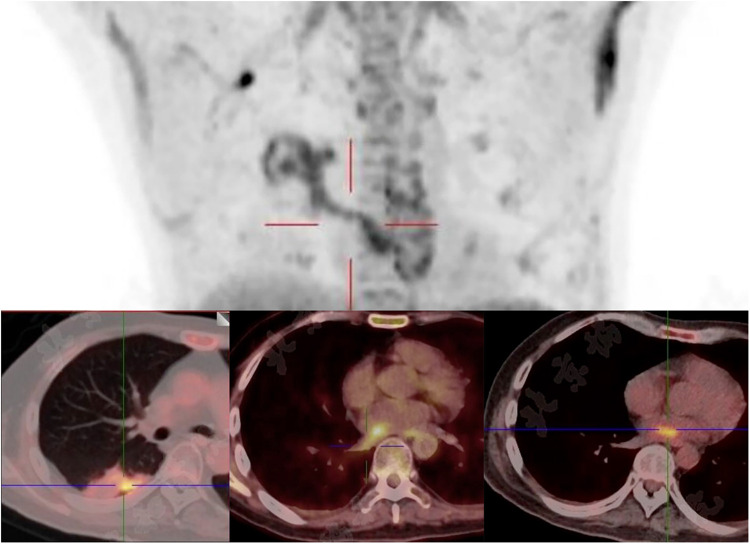
Focal infectious uptake was detected in the right lower lobe dorsal segment on PET/CT. The SUVmax is 5.0 in the pulmonary lesion, 5.3 in the left atiral lesion, and 4.9 in the RIPV.

**Figure 4:**
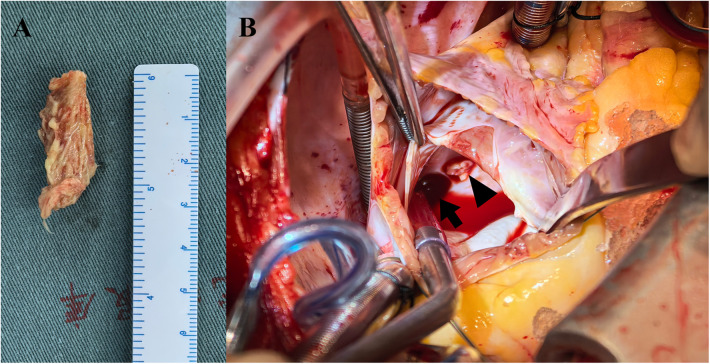
Mycelial vegetation within the left atrium **(A)** vegetation arising from a RIPV tributary (arrowhead), with the RIPV trunk indicated (arrow) **(B)**.

**Figure 5:**
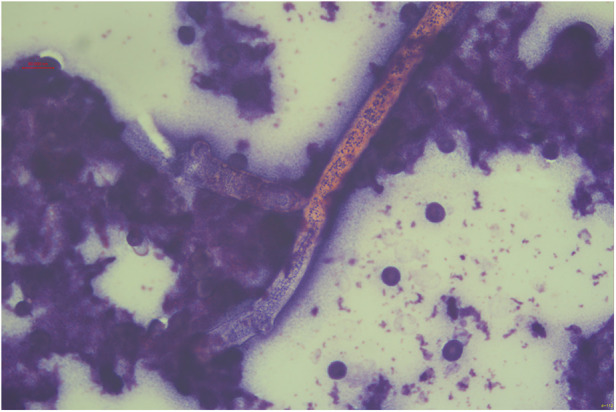
Fungal hyphae morphologically consistent with *V. volvacea* are observed under microscopy (×125 magnification, hematoxylin and eosin staining).

Given the multiple cardiac-source septic emboli, surgical resection of the left atrial vegetation was planned for this patient. Whole-body PET/CT 1 day before surgery showed a right lower lung lobe infectious focus spreading via the RIPV to the left atrium (Figure 3), no suspected intracranial infectious foci were noted. Preoperative labs showed pancytopenia: hemoglobin 75 g/L, neutrophils 3.44 × 10^9^/L, and platelets 26 × 10^9^/L. Perioperative risks were minimized by component transfusion. Intraoperatively, routine cardiopulmonary bypass was established via cannulation of the ascending aorta and superior/inferior vena cava. Through an atrial septal incision, the left atrium was explored, revealing a 4 × 1 cm hyphae-like vegetation (Figure 4) extending from the RIPV into the left atrial cavity, causing complete obstruction of the RIPV. After meticulous excision of the intracardiac portion, further exploration of the RIPV demonstrated the vegetation originating from its dorsal segmental tributary. The venous part of the vegetation was carefully dissected along the RIPV and its tributaries until reaching positions beyond instrument access. Procedures were performed within the pulmonary veins without injuring the endothelium, thereby eliminating the need for pulmonary vein isolation or reconstruction. Following resection, adequate backflow bleeding was confirmed from the RIPV and its tributaries. Total bypass time was 106 min and the aortic cross clamp time was 77 min. Histopathology of the vegetation confirmed *V. volvacea* endocarditis (Figure 5), further verified by *V. volvacea* DNA PCR.

Postoperative course was uneventful, the patient was extubated on postoperative day 1, discharged on postoperative day 4, and received liposomal amphotericin B combined with voriconazole for continued antifungal therapy at a primary care hospital. The trough concentration of liposomal amphotericin B is maintained at 1–3 mg/L. The patient's body temperature remained stable within the normal range. Repeat plasma mNGS demonstrated undetectable *V. volvacea* copies. Follow-up chest CT scan revealed a reduction in the size of the residual right pulmonary lesion (from 1.4 × 1.3 cm to 0.8 × 0.8 cm) 2 months postoperatively, and it was completely resolved at the 3-month postoperative follow-up. Intravenous antifungal therapy was discontinued at this point and transitioned to oral voriconazole. The patient is currently continuing oral voriconazole therapy, remains asymptomatic and feeling well about the treatments.

## Discussion

HSCT recipients face amplified infection risks due to factors such as central venous catheter placement, immunosuppressive therapy, and chemotherapy. The literature has documented two cases of Aspergillus endocarditis following HSCT. Both cases required histopathological confirmation and demonstrated clinical improvement after surgical debridement combined with long-term antifungal therapy (3, 4). Infections caused by *V. volvacea* nearly universally involve the lungs and brain (1, 5), manifesting as fever, confusion, headaches, and pulmonary infiltrates. Catastrophic complications (e.g., cerebral infarction, pulmonary embolism) often result from fungal emboli (1). Fungal endocarditis post-HSCT is exceedingly rare but carries 95% mortality (6). Only one case of *V. volvacea* endocarditis treated with cardiac surgery has been documented, with the patient succumbing 7 months postoperatively, due to an intracranial hemorrhage associated with a residual infectious lesion (7).

In the present case, immunosuppression post-HSCT may have contributed to the emergence of the incipient pulmonary fungal colonization, which wasn't timely identified and culminated in fungemia. During the fungemia phase, oral voriconazole therapy likely provided suboptimal drug exposure, contributing to progression from pulmonary fungal colonization to IFI and left atrial fungal endocarditis. After the diagnosis of IFI, multi-organ embolization highlighted the urgency of surgical intervention.

From fever onset through the surgical period, the patient remained persistently immunocompromised due to prior HSCT. Conventional infection biomarkers (e.g., total leukocyte count) had diminished clinical relevance for monitoring. During this critical phase, quantitative mNGS measurement of microbial genomic load served as an effective diagnostic and therapeutic monitoring tool. On the other hand, IFI may form new hematogenously disseminated foci. According to the literature, FDG-PET/CT demonstrates superior sensitivity over conventional CT in detecting disseminated IFI lesions (8). Consequently, comprehensive radiological screening—particularly whole-body PET/CT—holds paramount significance for diagnostic confirmation.

Preoperatively, trilineage cytopenia may stem from post-HSCT sequelae and constitutes elevated surgical risk. However, the administration of intensive intravenous antibiotic prophylaxis with targeted blood component replacement enables safe surgical execution. Surgical intervention confirmed the histopathological diagnosis of *V. volvacea* endocarditis and delineated its progression, while mitigating the risk of fungal vegetation embolism during subsequent antifungal therapy. Postoperatively, serial plasma mNGS and chest CT scan were employed for longitudinal surveillance of residual infectious lesion and fungemia. Oral antifungal therapy was deferred until complete radiographic resolution of pulmonary infiltrates to prevent late complications associated with residual infections. Furthermore, unlike the 2020 report (5), intracranial infectious foci were excluded for the present case via whole-body PET/CT. Given the absence of imaging evidence of residual infection at present, a good mid-term prognosis can be anticipated.

## Conclusion

IFIs post-HSCT represent a formidable clinical challenge, with fungal endocarditis being a rare yet lethal complication. We present a case of successfully managed *V. volvacea* endocarditis. This case highlights five critical determinants in this clinical phenomenon:

1. Heightened clinical vigilance toward emerging fungal pathogens;
2. Early initiation of aggressive intravenous antifungals at clinical suspicion;
3. Timely debridement for the endocarditis;
4. Extended postoperative antifungal consolidation;
5. Multimodal surveillance using both plasma mNGS and radiography.

## Limitations

This case report is limited by its focus on a single patient's treatment course and outcomes, which restricts generalizability. Additionally, the follow-up duration was relatively short (5 months).

## Data Availability

The original contributions presented in the study are included in the article/Supplementary Material, further inquiries can be directed to the corresponding author.
