# Correction: “Mushroom in the heart”: a Volvariella volvacea infective endocarditis case report

**DOI:** 10.3389/fcvm.2025.1745976

**Published:** 2025-11-25

**Authors:** Frontiers Production Office

**Affiliations:** Frontiers Media SA, Lausanne, Switzerland

**Keywords:** *Volvariella volvacea*, infective endocarditis, invasive fungal infections, allogeneic HSCT, fungal endocarditis surgery

The figures were in the wrong order in the PDF and HTML version of this paper. Figure 1 was located at the position of Figure 3, Figure 2 was located at the position of Figure 5, Figure 3 was located at the position of Figure 1 and Figure 5 was located at the position of Figure 2.

The order has now been corrected.

The original version of this article has been updated.

